# Impacts of conservation agriculture on soil structure and hydraulic properties of Malawian agricultural systems

**DOI:** 10.1016/j.still.2020.104639

**Published:** 2020-07

**Authors:** Samuel Eze, Andrew J. Dougill, Steven A. Banwart, Thirze D.G. Hermans, Ivy S. Ligowe, Christian Thierfelder

**Affiliations:** a School of Earth and Environment, Faculty of Environment, University of Leeds, LS2 9JT, Leeds, UK; b Department of Agricultural Research Services, Chitedze Research Station, P.O. Box 158, Lilongwe, Malawi; c CIMMYT, P.O. Box MP 163, Mount Pleasant, Harare, Zimbabwe

**Keywords:** Conservation agriculture, Soil structure, Soil water retention, Porosity, Dry spells, Climate resilience

## Abstract

Sub-Saharan Africa (SSA) faces climate change and food insecurity challenges, which require action to create resilient farming systems. Conservation agriculture (CA) is widely promoted across SSA but the impacts on key soil physical properties and functions such as soil structure and hydraulic properties that govern water storage and transmission are not well understood. The aim of this study was to assess the impacts of long term (10–12 years) maize-based CA on soil hydraulic conductivity, water retention and pore size distribution. Root zone (0–30 cm depth) soil total porosity, pore size distribution, saturated hydraulic conductivity (K_sat_) and plant available water capacity (PAWC) of conventional maize monocrop farming systems (CP) are compared with those of adjacent CA trials with either sole maize or maize intercrop/rotation with cowpea (*Vigna unguiculata* L.), pigeon pea (*Cajanus cajan* L.) or velvet bean (*Mucuna pruriens* L) in trial locations across central and southern Malawi. Results show that maize-based CA systems result in significant changes to soil hydraulic properties that correlate with improved soil structure. Results demonstrate increases of 5–15 % in total porosity, 0.06−0.22 cm/min in K_sat_, 3–7 % in fine pores for water storage and 3–6 % in PAWC. Maize monocrop CA had similar effect on the hydraulic properties as the maize-legume associations. The values of K_sat_ for CA systems were within optimum levels (0.03–0.3 cm/min) whereas PAWC was below optimum (<20 %). There was no significant build-up in soil organic matter (OM) in the CA systems. The results lead to a recommendation that crop residue management should be more pro-actively pursued in CA guidance from agricultural extension staff to increase soil OM levels, increase yields and enhance climate resilience of sub-Saharan African farming systems.

## Highlights

• Long-term (10–12 years) maize-based CA increased PAWC and K_sat_.
• K_sat_ was optimum (0.03–0.3 cm/min) in CA systems but PAWC was relatively low (<20 %).
• CA increased soil total porosity by 14–49 % and fine water storage pores by 33–171 %.
• There was a significant positive correlation between fine storage soil pores and PAWC.
• There was low OM build-up in the CA systems.

## Graphical abstract

**Figure:**
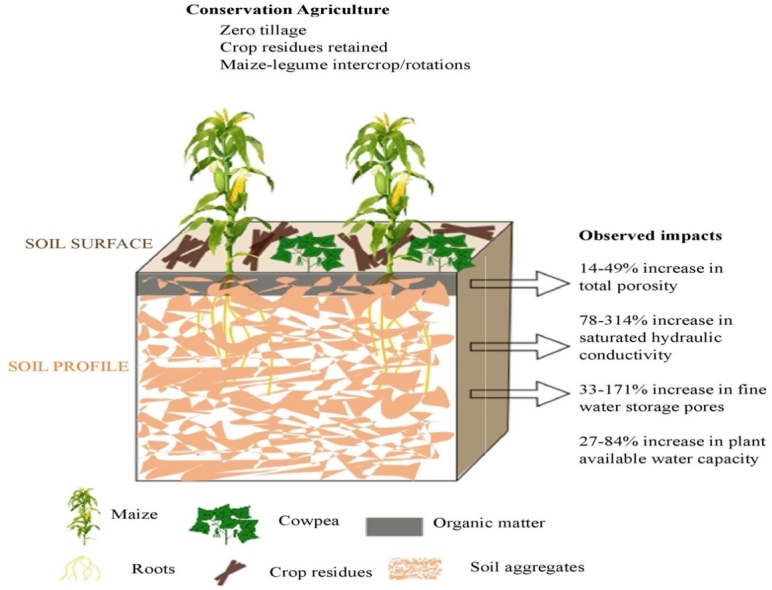

## 1 Introduction

Climate change, climate extremes, soil fertility decline and food insecurity are significant challenges facing sub-Saharan Africa (SSA), with 23 % of the population being undernourished and over 35 million people expected to be food insecure by 2050 (FAO and ECA, 2018). Adopting farming systems that are resilient to climate change and climate extremes is one important strategy for addressing these challenges (Altieri et al. (2015)). The climate resilience of farming systems depends on soil attributes such as structure, nutrient content, organic matter (OM) and biota (Lal, 2011; Cardoso et al. (2013)). Soil structure can be modified by land management (Bronick and Lal, 2005) and influences numerous soil processes and functions such as water and nutrient retention and transport, aeration, resistance to physical erosion, microbial activities and root growth (Banwart et al., 2019). Hence, understanding the structural characteristics of soils (e.g. pore size distribution and geometry, hydraulic conductivity and water retention capacity) under different agricultural systems provides insight into the resilience of such systems to changing environmental conditions.

This study focuses on Malawi, where over 80 % of the population depend on rain-fed agriculture with *circa* 60 % being food insecure (WFP, 2019). The dependence on rainfall makes the country vulnerable to severe events such as heat stress, dry spells and flooding, which have been associated with a significant decline in crop productivity and a deepening food security crisis (Thulu et al. (2017)). A significant deficit (-790,000 million tonnes) in maize (the main staple crop in Malawi) in the 2015/2016 cropping season (FIRP, 2016) was caused by the El Niño induced drought (Whitfield et al., 2019). The prevailing practice of preparing seed beds with hand hoes in the form of a ridge and furrow system with limited organic mulching, known as conventional practice (CP), has been shown to increase the negative response of maize to climate stress (Steward et al. (2018)). Maize-based CP systems are characterised by: the formation of a compact layer at about 25 cm below the soil surface which impedes water infiltration as well as root penetration and distribution within the rhizosphere (Materechera and Mloza-Banda, 1997); inefficient soil water conservation during dry periods (Thierfelder et al., 2013); disruption of continuous soil pores resulting in lower infiltration and transmission of rain water to plant roots (Thierfelder et al., 2005); increased susceptibility of soils to drying and moisture loss when ridges are not covered with mulch (Thierfelder and Wall, 2009); and the tendency for plant roots and associated mycorrhizal fungi to be disturbed and cut during weeding and reconstruction of ridges (Thierfelder et al., 2013).

Sustainable land management practices such as conservation agriculture (CA) with the potential to improve soil structural characteristics are advocated as strategies to buffer crop yields against climate stress. CA is a form of climate-smart agriculture characterised by minimal soil disturbance, permanent soil cover with organic materials and crop diversification (Lipper, 2010). Both government and non-governmental organizations encourage the adoption of CA by smallholder farmers in Malawi (Bell et al. (2018); NAIP, 2018) but there is still a wide knowledge gap on CA nationally especially with respect to hydrology and soil structure impacts (Dougill et al. (2017)).

Improvement in soil hydraulic properties such as increased water infiltration and transmission, soil moisture retention and plant available water capacity is perceived as the main mechanism underlying the enhanced maize yield of CA relative to CP under climate stress (Steward et al. (2018)). Although the connection between CA and an increase in infiltration is relatively well established (Ngwira et al. (2012); Thierfelder et al., 2013; TerAvest et al. (2015)), only few studies (see Table 1) have reported the impacts of CA on other critical soil hydraulic properties such as water retention and transmission. There is also no consistency in the reported effects of CA on soil water retention and transmission; on saturated hydraulic conductivity (K_sat_), field capacity (FC), permanent wilting point (PWP) and plant available water capacity (PAWC) (see Table 1). Another important soil hydraulic property that has received little research attention is pore size distribution. Understanding the soil pore architecture helps to explain the movement and storage of water and nutrients, the diversity of habitat for biota such as plant roots, soil fauna and microbial communities as well as the decomposition of organic matter (Negassa et al. (2015)). Soil pore architecture also influences the release of nutrient elements for crops and the development of anoxia which influences greenhouse gas emissions (Banwart et al., 2019).

**Table 1:** Reported short term effects of CA on soil hydraulic properties of soils with different particle size distribution in Malawi.

Location	Management	Duration (years)	Depth (cm)	Sand (%)	Clay (%)	BD (Mg/m^3^)	K_sat_ (cm/min)	FC (% vol.)	PWP (%vol.)	PAWC (%vol.)	Source
Balaka, southern Malawi (14°30′ - 15°20′S and 34°40′ - 35°30′S E; 625 masl)	Maize CA – no-till plus crop residue mulch	2	0−10	36^l^ (45)	43^h^ (37)	1.49^n^ (1.53)	174,000^l^ (201,000)	35.4^h^ (32.5)	23.2^h^ (20.9)	12.1^n^ (11.6)	Mloza-Banda et al., 2016
	Maize CA – no-till plus crop residue mulch	5	0−10	37^l^ (45)	43^h^ (38)	1.58^n^ (1.59)	184,800^n^ (189,000)	35.9^h^ (32.2)	23.7^n^ (21.3)	12.1^h^ (10.9)	Mloza-Banda et al., 2016
Central Malawi (13°29′ - 15°20′S and 33°14′ - 35°56′S E; 1100−1300 masl)	Maize CA – no-till plus crop residue mulch	2	0−10	50 (46)	37 (41)	1.34^l^ (1.51)	0.024^h^ (0.011)	19.3^n^ (14.7)	11.7^n^ (9.6)	7.6^n^ (5.1)	Mloza-Banda et al., 2014
	Maize CA – no-till plus crop residue mulch	4	0−10	53 (65)	35 (25)	1.62^n^ (1.47)	0.040^h^ (0.015)	24.6^n^ (17.6)	18.2^n^ (13.1)	6.4^n^ (4.5)	Mloza-Banda et al., 2014

BD = bulk density, K_sat_ = saturated hydraulic conductivity, FC = field capacity, PWP = permanent wilting point, PAWC = plant available water capacity, CA = conservation agriculture, vol. = volume, Values in parenthesis were recorded in plots under conventional practice, ^l^ = significantly lower, ^h^ = significantly higher, ^n^ = not significantly different. N.B. K_sat_ values 174,000, 201,000, 184,800 and 189,000 cm/min were converted from 29.0, 33.5, 30.8 and 31.5 m/s reported in Table 3 of Mloza-Banda et al. (2016).

The lack of consistency in the reported effects of CA on most soil hydraulic properties in Malawian maize-based CA systems may be partly due to the short duration of studies (typically 2–5 years), and the influence of site-specific characteristics such as soil texture. It is therefore necessary to investigate long term (at least 10 years) CA systems alongside CP systems with similar soil texture in order to detect real CA impacts on soil hydraulic properties that are critical for improved crop productivity and resilience.

This study investigated the impacts of long term (10–12 years) CA trials in central and southern Malawi on soil hydraulic properties at three trial locations. Specific objectives were:

i To investigate the impacts of CA practices on soil hydraulic conductivity and moisture retention;
ii To assess the impacts of CA on soil pore size distribution;
iii To compare the relationships between silt and clay particles, water retention and pore size distribution in CA and CP plots.

It was hypothesized that: 1) CA practices will increase soil hydraulic conductivity and water retention; 2) CA plots will have significantly higher volumes of storage pores than CP plots; and 3) There is a significant correlation between silt and clay particles, water retention and storage pores.

## 2 Methodology

### 2.1 Study area and experimental design

The study was carried out in three trial locations across central and southern Malawi (Table 2), where long term CA trials were established in smallholder farms in Lemu and Mwansambo, and at a government research station in Chitedze.

**Table 2:** Description of study sites.

Region	Site	Number of paired plots	Latitude (°)	Longitude (°)	Altitude (masl)	Soil type	Rainfall regime (mm)	Year CA started
Central	Chitedze	4	−13.298	34.129	1146	Chromic Luvisol	800−1000	2007
Central	Mwansambo	6	−13.306	34.118	652	Haplic Lixisols	1000−1300	2009
Southern	Lemu	6	−14.792	35.011	703	Chromic Luvisols	800−900	2007

Detailed descriptions of the study sites, experimental designs and management practices have been given in previous publications (e.g. Thierfelder et al., 2013; Ligowe et al., 2017; Steward et al., 2019). The CA systems were maize-based and consisted of maize monocrop, as well as maize intercrop or rotation with cowpea (*Vigna unguiculata* L.), pigeon pea (*Cajanus cajan* L.) and velvet bean (*Mucuna pruriens* L). In Chitedze, seven CA and one CP treatments were established on plots of 24m × 13.5m size with 18 rows of maize and laid out in a randomized complete block design with four replications. The treatments were:

1) Conventional practice (CP): Ridge and furrow system made with hand hoes, continuous maize monocrop with crop residues removed;
2) CA basin maize (CABM): No tillage system with planting on basins (0.15m × 0.15m x 0.15 m), continuous maize monocrop, crop residues retained on the soil surface;
3) CA direct maize (CADM): No tillage system with planting done by direct seeding with a dibble/pointed stick, continuous maize monocrop, crop residues retained on the soil surface;
4) CA cowpea rotation (CACR): No tillage system with planting done by direct seeding with a dibble/pointed stick, cowpea-maize-cowpea annual rotation, crop residues retained on the soil surface;
5) CA maize rotation (CAMR): No tillage system with planting done by direct seeding with a dibble/pointed stick, maize-cowpea-maize annual rotation, crop residues retained on the soil surface;
6) CA pigeon pea intercropping (CAPI): No tillage system with planting done by direct seeding with a dibble/pointed stick, maize-pigeon pea intercrop, crop residues retained on the soil surface;
7) CA cowpea intercropping (CACI): No tillage system with planting done by direct seeding with a dibble/pointed stick, maize-cowpea intercrop, crop residues retained on the soil surface;
8) CA velvet bean intercropping (CAVI): No tillage system with planting done by direct seeding with a dibble/pointed stick, maize-velvet bean intercrop, crop residues retained on the soil surface.

Weeds were controlled manually up to three times per season (depending on the level of weed infestation) with a hand hoe or by hand-picking in all the treatments. The CA treatments were initially sprayed with a pre-emergent herbicide (2.5 l/ha of glyphosate) prior to manual weeding. All the treatments also received 150 kg ha^−1^ of N-P-K (23-21−0 + 4S) fertilizer approximately two weeks after planting and 100 kg ha^−1^ urea approximately five weeks after planting based on local fertilizer recommendations.

In each of the on-farm trial sites in Lemu and Mwansambo, two CA and one CP treatments were replicated in six small-holder farms on plots of 3000 m^2^. The treatments were:

1) CP (as in Chitedze Research Station): weeding was done manually with hand hoe and carried out two or three times before the tasselling stage;
2) CADM: To control weeds, pre-emergence herbicide – a mixture of 2.5 L ha^−1^ glyphosate (N-(phosphono-methyl) glycine), Harness® (acetochlor (2-ethyl-6-methylphenyl-d11)) (Mwansambo) or 6 L ha^−1^ of Bullet® (25.4 % Alachlor (2-chloro-N-(2,6-diethylphenyl)-N-(methoxymethyl) acetamide) and 14.5 % atrazine (2-Chloro-4-ethylamino-6-isopropylamino-1,3,5-triazine)) was applied after planting. Subsequent weeding was done manually with a hand hoe.
3) CAPI in Lemu and CACI in Mwansambo: The legumes were planted between maize rows at in-row spacing of 40 cm (cowpea) or 50 cm (pigeon pea). Post-planting application of 2.5 L ha^−1^ glyphosate and subsequent manual weeding with a hand hoe were used to control weeds.

The CA and CP treatments in Lemu and Mwansambo were rotated annually with groundnut-pigeon pea intercrop. Sometimes, farmers incorporate crop residues in the ridges of the CP plots. All the plots received a uniform fertilizer application rate of 69 kg N ha^−1^ which was supplied as 150 kg N-P-K (23-21−0 + 4S) ha^-1^ at planting and 100 kg urea (46 % N) ha^−1^ at approximately three weeks after planting. The target population of 53,333 maize plants ha^−1^ was maintained in all the treatments at all the study sites by ensuring a plant spacing of 75 cm between rows and 25 cm within rows. At the end of the growing season, crop residues in each CA plot were retained and manually spread over the soil surface.

### 2.2 Sampling procedures

Soil samples were collected from all the study sites during the growing season in February 2019. Undisturbed soil cores (5 cm diameter x5 cm height) were taken from each treatment plot at four depths: 0−5 cm; 5−10 cm; 10−20 cm and 20−30 cm. The length of the sampling rings available at the time of sampling was 5 cm. Hence, undisturbed core samples were collected from 12.5 to 17.5 cm and 22.5–27.5 cm depths to represent 10−20 cm and 20−30 cm soil depths. This yielded a total of 272 soil cores. The soil cores were trimmed at both ends immediately after collection, covered with plastic caps and transferred to the Soil Physics laboratory of Chitedze Research Station, for the determination of bulk density, K_sat_, and water retention characteristics. Five additional soil samples were collected with an Edelman auger from each treatment plot at 0−5 cm, 5−10 cm, 10−20 cm and 20−30 cm depths, and bulked into a composite sample for determination of particle size distribution.

### 2.3 Laboratory analysis

Soil cores were covered at the lower end with a piece of muslin fabric and saturated gradually by capillary action in a plastic basin over a period of two to four days. K_sat_ measurements were then carried out using the constant-head method (Klute and Dirksen, 1986). The soil cores were re-saturated and water retention was measured at 0, -10, -33 and −1500 kPa matric potentials. Soil cores were subjected to -10 and −33 kPa matric potentials in a 5-bar pressure plate (1600 model, Soilmoisture Equipment, USA) and −1500 kPa matric potential in a 15-bar pressure plate (1500 model, Soilmoisture Equipment, USA) apparatus. Soil cores were allowed to drain at each pressure level until there was no change in weight. At the end of the moisture extraction at −1500 kPa, the cores were oven-dried at 105 °C for 24 h and the volumetric moisture content at each pressure level calculated as mass loss on drying. Bulk density was also calculated as the ratio of the weight of the oven-dried soil core to the volume of the core. The Bouyoucos Hydrometer method (Gee and Bauder, 1986) was used to determine the particle size distribution of soils. Total carbon in soil samples passed through a 0.5 mm sieve was analysed by combustion in an elemental analyser (Vario Micro Cube; Yeomans and Bremner, 1991).

### 2.4 Soil water retention curve and pore size classification

The parameters of the van Genuchten (1980) equation (Eq. 1) were fitted by statistical non-linear regression to the measured volumetric water contents at each value of matric potential.

(1) $$\theta =\;\theta_{r}\;+\;\frac{\theta_{s\;}-\theta_{r}}{{\left[1\;+\;{\left|\alpha \psi \right|}^{n}\right]}^{m}}$$

*θ* is the volumetric water content (m^3^/m^3^), *θ_r_* and *θ_s_* are residual and saturated soil volumetric water contents respectively (m^3^/m^3^), and *ψ* is the soil water matric potential (cm). The parameter *α* is the inverse of the air entry potential (cm^−1^), and *n* and *m* are dimensionless parameters associated with pore size distribution. The “Solver” function in Microsoft Office Excel (2016 version) was used to estimate the best fit parameter values for *θ_r_*, *α*, and *n* based on values that produced the smallest residual error between measured and calculated values for *θ*. The soil moisture retention parameters were derived for every site, management type and soil depth (see Table A1). Combining Eq. 1 with the Mualem (1976) restriction (Eq. 2) provided a good fit to the experimental data. Regression lines fitted through plots of predicted and measured water retention data had an average slope of 0.88, average coefficient of determination of 0.88 and root mean square value of 2.54.

(2) $$m=1-\frac{1}{n}$$

PAWC was calculated as the difference between volumetric water content at −33 kPa (FC) and volumetric water content at −1500 kPa (PWP). Air capacity (AC) which is an indication of soil aeration was calculated as the difference between θ_s_ and FC.

Effective pore sizes were determined from the predicted and measured soil water retention data using the Kelvin equation:

(3) $$d=\;\frac{4\gamma cos\alpha }{pgh}\;\;$$

Where *d* is the equivalent pore diameter (m), *h* is the matric potential (m), γ is the surface tension of water (72.75 mJ m^−2^), α is the pore-water contact angle (taken to be zero), *p* is water density (0.998 Mg m^-3^), and *g* is the gravitational acceleration (9.8 m s^−2^). Thus the diameter (d) of the smallest pore drained at a specific matric potential was calculated from Eq. 4 (Gregorich and Carter, 2007).

(4) $$d\;\left(\mu m\right)=\;\frac{297.5}{Matric\;potential\;\left(kPa\right)}$$

Four pore size classes determined were transmission pores (>60 μm effective diameter), coarse storage pores (10−60 μm effective diameter), fine storage pores (0.2−10 μm effective diameter) and residual pores (<0.2 μm effective diameter). The effective pore diameters used for the classification correspond to the matric potential values of -5, -30, and −1500 kPa. These pore classes were based on the terminologies used in previous pore classifications (e.g. Greenland, 1977; Hayashi et al. (2006)) and have different functions in relation to water transport: 1) Transmission pores hold water loosely such that it freely drains under gravity and is unavailable to plants; 2) Coarse storage pores hold water strongly enough not to drain under gravity and is easily available to plants; 3) Fine storage pores hold water with greater capillary force than coarse storage pores making it less available to plants; and 4) Residual pores hold water so tightly that it is unavailable to plants. Total porosity was assumed to be equal to the volumetric water content of the soil at saturation, which was calculated as mass loss on drying saturated soil cores at 105 °C to constant weight. Total porosity was not derived from measured bulk density (porosity = 1-[bulk density/particle density]) to avoid overestimation of porosity values as particle density was not measured. Using a constant value of particle density (e.g. 2.65 Mg/m^3^) to derive total porosity has been shown to overestimate the porosity value compared to that derived from measured particle density (Blanco-Canqui et al., 2006). This is because soil particle density depends on the composition of both the mineral and organic soil components and varies with management practices (Rühlmann et al., 2006).

### 2.5 Statistical analyses

Two-way analysis of variance was conducted for each of the three sites to compare the mean particle size distribution, bulk density, hydraulic conductivity, water retention and pore size distribution between the CA and CP plots and across depths. This was done after establishing the normality and homogeneity of variance of the data using the Shapiro-Wilk normality test and Levene’s test. Mean separation was conducted using the Tukey HSD post hoc test. The relationships between measured parameters particularly silt and clay content, hydraulic conductivity, water retention and pore size distribution were determined using Pearson’s product-moment correlation coefficient. The variability of the study sites based on measured soil characteristics was explored using principal component analysis (PCA). The significance of all the statistical tests were established at the 95 % confidence level. Mean comparison and separation were carried out with the software SPSS Statistics (version 25) whereas correlation and principal component analysis were performed in R Statistics (version 3.4.2).

## 3 Results

Across the three trial sites, land management had significant effects on K_sat_, PAWC, transmission pores, fine storage pores and residual pores (see Table 3, Table 4, Table 5, Table 6). The sites are characterised by sandy clay loam and sandy loam soils with clay contents ranging from 16 to 24% (see Tables A2-A3).

**Table 3:** Analysis of variance summary for measured soil hydraulic properties.

Parameter	Source of variation	Chitedze		Mwansambo		Lemu	
		F-value	p-value	F-value	p-value	F-value	p-value
Total porosity (%)	Management (Mgt)	7.650	<0.001	4.952	0.010	1.476	0.237
	Depth	0.050	0.985	0.372	0.774	1.748	0.167
	Mgt x Depth	0.507	0.961	0.267	0.950	2.433	0.036
Transmission pores (%)	Mgt	10.979	<0.001	27.778	<0.001	74.278	<0.001
	Depth	5.399	0.002	7.199	<0.001	3.032	0.036
	Mgt x Depth	6.123	<0.001	29.417	<0.001	16.239	<0.001
Coarse storage pores (%)	Mgt	0.957	0.467	0.829	0.441	2.919	0.062
	Depth	2.439	0.069	9.975	<0.001	4.665	0.005
	Mgt x Depth	1.617	0.061	4.484	0.001	4.027	0.002
Fine storage pore (%)	Mgt	19.073	<0.001	19.567	<0.001	151.114	<0.001
	Depth	0.800	0.497	6.937	<0.001	4.533	0.006
	Mgt x Depth	1.604	0.064	1.025	0.418	11.830	<0.001
Residual pores (%)	Mgt	11.484	<0.001	44.042	<0.001	378.041	<0.001
	Depth	1.856	0.142	1.341	0.269	67.244	<0.001
	Mgt x Depth	0.748	0.773	8.270	<0.001	33.447	<0.001
Saturated hydraulic conductivity (cm/min)	Mgt	7.966	<0.001	26.013	<0.001	8.502	0.001
	Depth	17.2	<0.001	27.897	<0.001	26.381	<0.001
	Mgt x Depth	2.781	<0.001	8.831	<0.001	0.993	0.438
Plant available water capacity (%)	Mgt	10.477	<0.001	39.754	<0.001	76.072	<0.001
	Depth	0.505	0.680	1.813	0.154	2.706	0.053
	Mgt x Depth	0.791	0.724	0.080	0.998	4.104	0.002
Air exchange capacity (%)	Mgt	0.965	0.461	2.712	0.075	9.859	<0.001
	Depth	0.736	0.533	0.976	0.410	0.241	0.868
	Mgt x Depth	0.896	0.596	1.128	0.357	1.965	0.085

**Table 4:** Percentage change in soil hydraulic properties in CA plots relative to CP plots.

Site	Management	Total porosity (%)	Transmission pores (%)	Fine storage pores (%)	Residual pores (%)	K_sat_ (%)	PAWC (%)
Chitedze	CABM	45*	35*	110*	47*	229*	69*
	CADM	49*	43*	112*	57*	214*	69*
	CACR	47*	66*	85*	54*	314*	60*
	CAMR	34*	29	91*	36*	271*	48*
	CAPI	29*	10	94*	31*	157*	54*
	CACI	45*	57*	96*	45*	243*	62*
	CAVI	42*	2	129*	45*	243*	77*
Mwansambo	CADM	6	−40*	33*	17*	450*	27*
	CACI	14*	−29*	43*	22*	300*	41*
Lemu	CADM	4	−53*	94*	64*	44	65*
	CAPI	9	−53*	171*	62*	78*	84*

= significant change at 5% probability level, K_sat_ = saturated hydraulic conductivity, PAWC = plant available water capacity, CA = conservation agriculture (no tillage, direct seeding with dibble stick and crop residues retained on the soil surface), BM = continuous maize monocrop planted on basins (0.15m × 0.15m x 0.15 m), DM = continuous maize monocrop with planting done by direct seeding with a dibble/pointed stick, CR = cowpea-maize-cowpea annual rotation, MR = maize-cowpea-maize annual rotation, PI = maize-pigeon pea intercrop, CI = maize-cowpea intercrop, VI = maize-velvet bean intercrop. All the treatments in Lemu and Mwansambo were rotated annually with groundnut-pigeon pea intercrop.

**Table 5:** Mean ± standard deviation of saturated hydraulic conductivity and plant available water capacity across land management types, soil depths and sites.

Depth (cm)	Management type	Saturated hydraulic conductivity (cm/min)			Plant available water capacity (%)		
		Chitedze	Mwansambo	Lemu	Chitedze	Mwansambo	Lemu
0−5	CP	0.110 ± 0.03a	0.016 ± 0.00a	0.201 ± 0.13a	7.74 ± 1.0a	10.53 ± 0.6a	5.12 ± 0.6a
	CABM	0.330 ± 0.12ab			13.70 ± 2.2b		
	CADM	0.200 ± 0.07a	0.210 ± 0.11b	0.236 ± 0.04a	15.08 ± 1.3b	13.26 ± 3.0ab	9.40 ± 1.1b
	CACR	0.570 ± 0.23b			13.20 ± 1.8ab		
	CAMR	0.280 ± 0.05a			11.57 ± 3.0ab		
	CAPI	0.160 ± 0.05a		0.271 ± 0.05a	13.28 ± 4.0ab		10.13 ± 1.0b
	CACI	0.250 ± 0.02a	0.234 ± 0.05b		13.24 ± 3.0ab	14.61 ± 2.1b	
	CAVI	0.340 ± 0.18ab			13.19 ± 2.0ab		
5−10	CP	0.070 ± 0.00a	0.016 ± 0.01a	0.043 ± 0.04a	7.70 ± 1.0a	9.70 ± 1.4a	5.15 ± 2.2a
	CABM	0.310 ± 0.10ab			12.94 ± 2.7ab		
	CADM	0.300 ± 0.07ab	0.053 ± 0.02b	0.104 ± 0.07a	13.36 ± 2.5ab	12.27 ± 0.8b	7.19 ± 0.8a
	CACR	0.300 ± 0.19ab			12.26 ± 1.2ab		
	CAMR	0.420 ± 0.27b			11.90 ± 4.0ab		
	CAPI	0.180 ± 0.02ab		0.095 ± 0.07a	10.38 ± 1.4ab		10.69 ± 1.8b
	CACI	0.350 ± 0.02ab	0.058 ± 0.02b		12.79 ± 2.4ab	13.89 ± 1.3b	
	CAVI	0.230 ± 0.07ab			16.22 ± 3.5b		
10−20	CP	0.060 ± 0.01a	0.017 ± 0.01a	0.041 ± 0.03a	7.73 ± 0.4a	9.75 ± 1.0a	5.40 ± 1.3a
	CABM	0.150 ± 0.02ab			12.47 ± 1.4b		
	CADM	0.210 ± 0.10b	0.052 ± 0.06a	0.062 ± 0.06a	12.15 ± 1.5ab	12.12 ± 0.6	10.55 ± 1.4b
	CACR	0.130 ± 0.02ab			12.42 ± 1.8b		
	CAMR	0.170 ± 0.07ab			10.97 ± 2.2ab		
	CAPI	0.170 ± 0.03ab		0.164 ± 0.02b	14.05 ± 2.6b		9.42 ± 1.7b
	CACI	0.190 ± 0.09ab	0.018 ± 0.01a		12.85 ± 1.3b	13.70 ± 2.4	
	CAVI	0.220 ± 0.04b			12.85 ± 3.3b		
20−30	CP	0.050 ± 0.02a	0.013 ± 0.00a	0.057 ± 0.03a	8.60 ± 0.1a	9.43 ± 0.8a	5.52 ± 1.2a
	CABM	0.150 ± 0.02b			14.52 ± 3.5b		
	CADM	0.150 ± 0.02ab	0.114 ± 0.07b	0.099 ± 0.03a	13.22 ± 1.0ab	12.35 ± 0.5b	7.83 ± 0.5b
	CACR	0.150 ± 0.02ab			12.83 ± 1.0ab		
	CAMR	0.190 ± 0.05b			12.43 ± 3.2ab		
	CAPI	0.190 ± 0.07b		0.107 ± 0.08a	11.16 ± 2.0ab		8.68 ± 1.1b
	CACI	0.160 ± 0.06b	0.023 ± 0.02a		12.51 ± 3.1ab	13.22 ± 2.1b	
	CAVI	0.160 ± 0.04b			14.10 ± 2.7ab		

Values followed by different letters in the same column and within the same depth differed significantly from each other at 5% probability level. CA = conservation agriculture (no tillage, direct seeding with dibble stick and crop residues retained on the soil surface), BM = continuous maize monocrop planted on basins (0.15m × 0.15m x 0.15 m), DM = continuous maize monocrop with planting done by direct seeding with a dibble/pointed stick, CR = cowpea-maize-cowpea annual rotation, MR = maize-cowpea-maize annual rotation, PI = maize-pigeon pea intercrop, CI = maize-cowpea intercrop, VI = maize-velvet bean intercrop. All the treatments in Lemu and Mwansambo were rotated annually with groundnut-pigeon pea intercrop.

**Table 6:** Mean ± standard deviation of soil pores across land management (Mgt) types, soil depths and sites.

Depth (cm)	Mgt type	Transmission pores (%)			Fine storage pores (%)			Residual pores (%)		
		Chitedze	Mwansambo	Lemu	Chitedze	Mwansambo	Lemu	Chitedze	Mwansambo	Lemu
0−5	CP	6.70 ± 2.2ab	4.52 ± 0.9a	19.37 ± 4.3a	5.23 ± 1.7a	9.01 ± 1.8a	3.49 ± 0.8a	12.27 ± 0.5a	17.71 ± 3.0a	8.31 ± 0.2a
	CABM	5.08 ± 0.7ab			12.10 ± 1.6b			19.21 ± 2.5b		
	CADM	7.31 ± 0.3ab	3.07 ± 0.4b	7.05 ± 0.9b	13.28 ± 0.5b	11.61 ± 1.3b	8.57 ± 1.0b	21.33 ± 0.8b	19.99 ± 2.1a	17.66 ± 0.7b
	CACR	6.37 ± 1.2ab			11.01 ± 2.0b			19.48 ± 3.0b		
	CAMR	4.75 ± 1.2a			10.31 ± 2.7b			17.56 ± 4.5ab		
	CAPI	6.74 ± 1.2ab		7.51 ± 1.1b	11.46 ± 2.1b		8.50 ± 1.2b	17.56 ± 3.1ab		17.19 ± 1.0b
	CACI	6.59 ± 1.2ab	3.16 ± 0.4b		11.71 ± 2.1b	12.61 ± 1.5b		20.31 ± 3.6b	20.74 ± 1.7a	
	CAVI	7.70 ± 1.1b			11.09 ± 1.6b			19.59 ± 2.8b		
5−10	CP	7.01 ± 2.7ab	6.67 ± 1.7a	19.52 ± 2.7a	3.35 ± 1.3a	6.33 ± 1.6a	4.07 ± 0.6a	13.18 ± 0.2a	19.02 ± 0.9a	8.40 ± 0.1a
	CABM	9.49 ± 1.5ab			11.09 ± 1.8bc			18.10 ± 2.9a		
	CADM	8.16 ± 0.8ab	3.70 ± 0.7b	7.92 ± 3.5b	12.19 ± 1.1bc	9.59 ± 1.7b	4.10 ± 1.8a	17.58 ± 1.7a	18.71 ± 0.9a	11.55 ± 0.2b
	CACR	10.82 ± 2.2b			9.42 ± 1.9b			18.50 ± 2.0a		
	CAMR	6.36 ± 1.5a			10.58 ± 2.5bc			16.73 ± 3.9a		
	CAPI	5.82 ± 1.6a		4.92 ± 0.6b	9.13 ± 2.6b		10.38 ± 1.3b	15.05 ± 3.2a		14.33 ± 1.8c
	CACI	7.66 ± 1.7ab	5.13 ± 0.7ab		10.59 ± 2.3bc	9.67 ± 1.4b		18.57 ± 3.8a	21.08 ± 0.5b	
	CAVI	6.35 ± 0.2a			14.17 ± 0.5c			18.81 ± 0.6a		
10−20	CP	3.53 ± 1.0a	2.30 ± 0.9a	10.01 ± 6.0a	5.96 ± 1.7a	8.11 ± 3.2a	3.30 ± 2.0a	12.42 ± 0.4a	18.38 ± 0.2a	9.66 ± 0.1a
	CABM	8.68 ± 1.4b			10.67 ± 1.8b			18.15 ± 2.8ab	20.97 ± 0.5b	17.67 ± 0.5b
	CADM	11.08 ± 1.1b	4.78 ± 0.8b	4.12 ± 0.4b	10.45 ± 1.1b	8.53 ± 1.5a	8.12 ± 0.8b	18.64 ± 1.9b		
	CACR	10.27 ± 2.0b			9.84 ± 1.9ab			18.68 ± 3.6b		
	CAMR	9.45 ± 1.9b			10.03 ± 2.0ab			15.85 ± 1.7ab		
	CAPI	3.39 ± 0.7a		13.82 ± 1.7a	12.77 ± 2.5b		8.41 ± 1.0b	17.59 ± 3.4ab		15.55 ± 1.7c
	CACI	11.55 ± 1.6b	5.84 ± 2.1b		10.85 ± 1.5b	9.86 ± 3.6a		18.14 ± 2.4ab	20.68 ± 2.2b	
	CAVI	4.44 ± 0.9a			11.77 ± 2.4b			16.91 ± 3.4ab		
20−30	CP	4.85 ± 1.1ab	10.00 ± 2.2a	18.18 ± 3.9a	7.46 ± 1.7a	7.40 ± 1.6a	2.57 ± 0.5 a	12.29 ± 0.2a	14.09 ± 0.7a	10.07 ± 0.3a
	CABM	6.51 ± 1.5ab			12.25 ± 2.8ab			18.23 ± 2.9bc		
	CADM	5.10 ± 0.6ab	2.45 ± 0.2b	12.65 ± 2.6b	10.70 ± 1.3ab	11.15 ± 0.7b	5.09 ± 1.0b	21.39 ± 0.9c	21.08 ± 0.6b	12.76 ± 0.4b
	CACR	9.09 ± 0.4a			10.49 ± 0.5ab			20.37 ± 0.9bc		
	CAMR	7.91 ± 1.8ab			11.04 ± 2.5ab			17.95 ± 3.8abc		
	CAPI	8.40 ± 4.8ab		5.60 ± 0.2c	9.33 ± 2.8ab		8.98 ± 0.3c	15.30 ± 3.4ab		12.15 ± 0.3c
	CACI	8.88 ± 2.4ab	2.49 ± 0.2b		9.97 ± 2.7ab	11.89 ± 0.7b		15.76 ± 2.7abc	21.94 ± 1.3b	
	CAVI	3.96 ± 0.6b			13.26 ± 1.9b			17.50 ± 2.4abc		

Values followed by different letters in the same column and within the same depth differed significantly from each other at 5% probability level. CA = conservation agriculture (no tillage, direct seeding with dibble stick and crop residues retained on the soil surface), BM = continuous maize monocrop planted on basins (0.15m × 0.15m x 0.15 m), DM = continuous maize monocrop with planting done by direct seeding with a dibble/pointed stick, CR = cowpea-maize-cowpea annual rotation, MR = maize-cowpea-maize annual rotation, PI = maize-pigeon pea intercrop, CI = maize-cowpea intercrop, VI = maize-velvet bean intercrop. All the treatments in Lemu and Mwansambo were rotated annually with groundnut-pigeon pea intercrop.

### 3.1 Saturated hydraulic conductivity and plant available water capacity

The CA plots had significantly higher (p < 0.05) K_sat_ and PAWC than the CP plots in all of the three sites (Table 4, Table 5). Across the three sites, the surface soil layers had significantly higher K_sat_ values than the lower soil layers (Table 5). There was no significant difference in PAWC between the surface and sub-surface soil layers in all the three sites. The interactive effects of land management and soil depth on K_sat_ were statistically significant only in Chitedze and Mwansambo (Table 5).

### 3.2 Soil pore size distribution

Total porosity increased in all the CA treatments relative to CP but this was only statistically significant in Chitedze and in CA with cowpea intercrop treatment in Mwansambo (Table 4 and Table A4). The transmission pores in Chitedze were higher in all the CA plots (except CA with cowpea intercrop) but this was statistically significant only at 10−20 cm soil layer (Table 6). The CA plots in both Mwansambo and Lemu had significantly lower volume of transmission pores than their corresponding CP plots across all depths. The volume of fine storage pores and residual pores in the three study sites were significantly higher in the CA plots than corresponding CP plots.

### 3.3 Correlation between silt and clay, soil water retention and pore size distribution

The relationships between silt and clay (s + c), PAWC and pore size distribution differed between CP and CA plots and between the three sites (Figures A1−3). In the CP plots, there was no significant correlation between s + c, PAWC and soil pores in Mwansambo and Lemu whereas a significant negative correlation exist between s + c and PAWC in Chitedze. In the CA plots of the three sites, there were significant positive correlations between fine storage pores and PAWC, and between residual pores and PAWC. The correlation between s + c and K_sat_ in CA plots was negative in all the sites but was statistically significant only in Chitedze and Mwansambo (Figures A1−3). In addition, transmission pores in the Mwansambo CA plots had a significant negative correlation with s + c.

## 4 Discussion

CA practices across central and southern Malawi improved the soil’s capacity to retain and transmit water within the root zone (0−30 cm depth). These results support the first hypothesis of our study which states that CA practices will increase soil hydraulic conductivity and water retention. The observed positive effects of CA on K_sat_ is consistent with the findings of a previous study in central Malawi (Mloza-Banda et al. (2016)) where short term (<5 years) CA practices increased K_sat_ to a maximum value of 0.04 cm/min. The higher values recorded in our study suggest that the duration of management is an important factor in realising the full benefits of CA to improve soil drainage. The CA-induced increase in transmission pores were observed in only one site (Chitedze), however, total volume of pores increased in all the three sites and this partly explains the higher K_sat_ observed in all the CA plots. The increase in K_sat_ may have been a result of stable pore configuration and connectivity (Bhattacharyya et al. (2006)) in CA plots. Since soil disturbance in CA plots is much reduced, there is a tendency for the development of a more stable soil structure with greater pore volume and pore connectivity than CP plots where tillage practices lead to the disruption of pore structure (Azooz and Arshad, 1996).

Across the three sites and regardless of the type of CA practised, K_sat_ values were within the range of values (0.03−0.3 cm/min) considered to be ideal for rapid infiltration and redistribution of plant-available water needed for optimum crop growth (Reynolds et al. (2003)) whereas the K_sat_ values of the CP plots were on the border of the lower critical limit (0.03 cm/min). This optimum K_sat_ which indicates adequate water movement within the root zone and ease of nutrient supply to plants, likely contributes to the increase in crop yield in CA plots reported by Thierfelder et al. (2013). It is however important to note that optimum K_sat_ does not necessarily mean that optimum levels of water and nutrients are always available for the crops as this depends on PAWC. In this study, CA improved PAWC with a range of values (9–14 %) greater than those (6–12 %) reported previously (Mloza‐Banda et al. (2014); (2016)) in CA fields in central and southern Malawi. Despite this relative increase in PAWC attributable to greater storage pores, the values are still below 20 %, and the soil moisture condition is considered highly susceptible to dry spells and limited for maximum root growth (Cockroft and Olsson, 1997).

It is known that OM plays an important role in improving soil structure. For example, OM additions increase total soil porosity by helping to bind soil particles into stable aggregates (Luna et al. (2018)) that results in both greater inter-aggregate and intra-aggregate pore volume. An earlier study in Mwansambo and Lemu showed that there was no significant increase in the soil OM content of the CA plots relative to CP after five-six years of establishment (Cheesman et al. (2016)). The soil OM status has not changed significantly after 10–12 years (Table A5) and other fine soil particles such as silt and clay content of CA and CP plots were similar (Table A3). Thus, the soil structural improvements observed in this study can be attributed to the influence of minimal soil disturbance. Previous studies have shown that minimum tillage is associated with an increase in the concentration of labile organic carbon (OC) pools and the formation of macro-aggregates (Bhattacharyya et al., 2012) which improve soil pore architecture. The presence of organic mulch on the surface of CA soils could reduce surface sealing, ponding and runoff generation (Prosdocimi et al. (2016)) thereby enhancing water infiltration and transmission within the root zone. Mulch cover also reduces the impact and shear forces of rainfall, wind and surface water flow on surface soil particles, thus reducing the incipient dislodging of particles that initiates physical erosion and sealing of soil pores (Thierfelder et al., 2005). Further improvements in soil structure and associated benefits may require more years of CA practices to enhance the OM status of the soil. Ensuring that farmers do not burn crop residues or remove the residues from the CA fields for other use such as livestock feed or fuel will also help to further increase OM inputs. A full assessment of carbon (C) balance in the CA systems is necessary in order to identify all the pathways of OM loss and take appropriate measures to minimise the losses. The distribution of OM within the soil matrix is another area that merits further research.

Improving the water retention of soils may help to explain the gap that still exists between actual observed maize yields (<10 Mg/ha) in CA fields (Thierfelder et al., 2013; 2015) and potential/attainable yields of up to 12 Mg/ha (Tamene et al. (2016)) in Malawi. Although there is a concern of declining nutrient status in the agricultural soils in Malawi (Omuto and Vargas, 2018) which can be a major contributor to the maize yield gap, improving the water retention of the soils through proper OM management will minimise nutrient leaching.

Minimum soil disturbance through zero tillage and mulching with crop residues were the two components of CA that seemed to exert significant influence on soil hydraulic properties. Crop diversification did not show any consistent effect on the hydraulic properties investigated in this study. For example, the effect of CA with maize monocrop on soil total porosity, storage pores, water transmission and retention was either the same or higher than the effects observed when maize was intercropped or rotated with cowpea, pigeon pea or velvet bean. The impacts of CA on soil hydraulic properties of on-farm trial sites that were fully rotated annually with groundnut-pigeon pea intercrop also did not differ from the impacts in Chitedze research station. This highlights the need to focus more on OM addition through proper crop residue management as a means of increasing the water retention of the soils. Crop diversification should not be overlooked as other benefits of maize-legume intercrop or rotation such as added income to farmers, diet diversification, pest control and fertility management are necessary for the resilience of the CA system to climate stress and food security. Ngwira et al. (2012) reported that maize-legume association in the CA plots was an added benefit to the system as the presence of legumes did not affect maize yields. Greater population of earthworms in CA systems with maize-cowpea rotations have also been reported (Ligowe et al., 2017) as an indication of higher OM accumulation.

## 5 Conclusion

The results of this study showed that 10–12 years of maize-based CA systems (either as maize monocrop or maize intercrop or rotation with cowpea, pigeon pea or velvet bean) in three trial sites across central and southern Malawi improved soil hydraulic properties including soil water storage pores, water transmission and retention within the root zone (0−30 cm depth). Whereas the water transmission capacity of the CA systems was optimum, the water retention was below optimum. The soil structural improvements observed were attributed to minimal soil disturbance as there was insignificant soil OM build-up. This highlights the need for improved crop residue management to maintain permanent soil cover, as an effective strategy for improving the water retention of the soils for greater crop productivity and resilience to environmental change. It is recommended that further studies should assess the carbon balance of the CA systems in order to identify pathways of OM loss and means of minimising such losses. Notwithstanding the need for improved OM management, the results of this study indicate that CA has the potential to increase the capacity of agricultural systems to store antecedent water and make it available to plants during dry spells.

## Declaration of Competing Interest

The authors declare that they have no known competing financial interests or personal relationships that could have appeared to influence the work reported in this paper.
