# Supplementary material for: Impacts of conservation agriculture on soil structure and hydraulic properties of Malawian agricultural systems
Source: Soil Tillage Res. 2020 Jul;201:104639. doi: 10.1016/j.still.2020.104639 (PMC7233133; doi:10.1016/j.still.2020.104639)
Supplement: Supplementary file 2 [file mmc2.docx]

**Supplementary Data**

Table A1: Soil water retention curve parameters.

| Site | Depth | Treatment | Θ_s_ (%) | Θ_r_ (%) | m | n | α (kPa^-1^) |
| --- | --- | --- | --- | --- | --- | --- | --- |
| Chitedze | 0-5 | 1 | 29.91 | 10.64 | 0.2696 | 1.3691 | 0.5382 |
|  |  | 2 | 42.92 | 0.00 | 0.1124 | 1.1266 | 0.3807 |
|  |  | 3 | 49.50 | 0.00 | 0.1110 | 1.1248 | 0.5651 |
|  |  | 4 | 43.27 | 2.76 | 0.1154 | 1.1304 | 0.5911 |
|  |  | 5 | 38.23 | 0.00 | 0.1068 | 1.1195 | 0.4481 |
|  |  | 6 | 42.46 | 0.00 | 0.1146 | 1.1295 | 0.6095 |
|  |  | 7 | 45.23 | 0.00 | 0.1051 | 1.1174 | 0.6098 |
|  |  | 8 | 44.85 | 0.00 | 0.1034 | 1.1154 | 0.8747 |
|  | 5-10 | 1 | 29.42 | 12.78 | 0.3653 | 1.5756 | 0.4407 |
|  |  | 2 | 45.44 | 0.00 | 0.1092 | 1.1226 | 1.2094 |
|  |  | 3 | 45.31 | 0.00 | 0.1194 | 1.1355 | 0.7186 |
|  |  | 4 | 45.44 | 8.96 | 0.1497 | 1.1761 | 1.3552 |
|  |  | 5 | 39.84 | 0.00 | 0.1122 | 1.1263 | 0.6414 |
|  |  | 6 | 35.69 | 3.72 | 0.1321 | 1.1522 | 0.6053 |
|  |  | 7 | 43.12 | 1.09 | 0.1089 | 1.1218 | 0.8960 |
|  |  | 8 | 47.47 | 0.85 | 0.1309 | 1.1507 | 0.3745 |
|  | 10-20 | 1 | 28.23 | 11.17 | 0.3112 | 1.4519 | 0.2141 |
|  |  | 2 | 43.99 | 1.00 | 0.1105 | 1.1242 | 1.0861 |
|  |  | 3 | 46.53 | 0.00 | 0.1023 | 1.1140 | 2.0334 |
|  |  | 4 | 44.75 | 0.73 | 0.1007 | 1.1120 | 2.0052 |
|  |  | 5 | 42.66 | 7.28 | 0.1659 | 1.1989 | 0.8288 |
|  |  | 6 | 39.96 | 0.00 | 0.1253 | 1.1433 | 0.2046 |
|  |  | 7 | 47.24 | 0.00 | 0.1072 | 1.1201 | 1.9291 |
|  |  | 8 | 39.43 | 0.00 | 0.1207 | 1.1373 | 0.3167 |
|  | 20-30 | 1 | 34.59 | 11.41 | 0.3673 | 1.5806 | 0.1863 |
|  |  | 2 | 44.65 | 5.39 | 0.1476 | 1.1731 | 0.4240 |
|  |  | 3 | 44.49 | 14.05 | 0.1890 | 1.2330 | 0.2989 |
|  |  | 4 | 46.17 | 1.01 | 0.1000 | 1.1111 | 1.3698 |
|  |  | 5 | 43.55 | 1.04 | 0.1144 | 1.1292 | 0.8384 |
|  |  | 6 | 39.48 | 3.90 | 0.1328 | 1.1531 | 4.1341 |
|  |  | 7 | 41.52 | 5.76 | 0.1509 | 1.1778 | 0.8696 |
|  |  | 8 | 41.49 | 0.00 | 0.1285 | 1.1475 | 0.2316 |
| Mwansambo | 0-5 | 1 | 36.23 | 2.38 | 0.1067 | 1.1195 | 0.5060 |
|  |  | 2 | 40.17 | 1.67 | 0.1141 | 1.1288 | 0.2124 |
|  |  | 3 | 42.78 | 6.39 | 0.1423 | 1.1659 | 0.1818 |
|  | 5-10 | 1 | 37.40 | 15.45 | 0.2075 | 1.2618 | 0.6861 |
|  |  | 2 | 38.78 | 13.96 | 0.2233 | 1.2876 | 0.2092 |
|  |  | 3 | 44.34 | 17.81 | 0.2626 | 1.3561 | 0.2379 |
|  | 10-20 | 1 | 39.30 | 17.95 | 0.4365 | 1.7746 | 0.1010 |
|  |  | 2 | 41.76 | 17.98 | 0.2588 | 1.3491 | 0.2538 |
|  |  | 3 | 43.76 | 14.79 | 0.2026 | 1.2541 | 0.3529 |
|  | 20-30 | 1 | 38.59 | 10.81 | 0.2325 | 1.3029 | 0.7728 |
|  |  | 2 | 40.40 | 12.20 | 0.1769 | 1.2149 | 0.1444 |
|  |  | 3 | 41.42 | 0.93 | 0.1061 | 1.1187 | 0.1674 |
| Lemu | 0-5 | 1 | 35.35 | 7.20 | 0.2661 | 1.3627 | 4.9225 |
|  |  | 2 | 39.93 | 12.00 | 0.1917 | 1.2372 | 0.5581 |
|  |  | 3 | 39.40 | 10.12 | 0.1687 | 1.2029 | 0.7364 |
|  | 5-10 | 1 | 37.91 | 7.54 | 0.3081 | 1.4453 | 1.9672 |
|  |  | 2 | 31.73 | 11.20 | 0.3944 | 1.6513 | 0.3435 |
|  |  | 3 | 35.57 | 0.00 | 0.1235 | 1.1409 | 0.4216 |
|  | 10-20 | 1 | 31.51 | 9.48 | 0.4304 | 1.7555 | 0.3800 |
|  |  | 2 | 35.87 | 13.10 | 0.2092 | 1.2646 | 0.2893 |
|  |  | 3 | 43.09 | 1.37 | 0.1064 | 1.1191 | 5.7379 |
|  | 20-30 | 1 | 33.36 | 8.80 | 0.2204 | 1.2828 | 23.4519 |
|  |  | 2 | 36.21 | 10.99 | 0.2575 | 1.3469 | 1.4139 |
|  |  | 3 | 32.17 | 0.00 | 0.1248 | 1.1426 | 0.6188 |

Table A2: Analysis of variance summary for measured soil texture and bulk density.

| Parameter | Source of variation | Chitedze | | Mwansambo | | Lemu | |
| --- | --- | --- | --- | --- | --- | --- | --- |
|  |  | F-value | p-value | F-value | p-value | F-value | p-value |
| Sand (%) | Management (Mgt) | 1.06 | 0.397 | 6.609 | 0.003 | 0.327 | 0.723 |
|  | Depth | 5.06 | 0.003 | 17.446 | <0.001 | 1.324 | 0.275 |
|  | Mgt x Depth | 0.63 | 0.885 | 0.194 | 0.977 | 0.063 | 0.999 |
| Silt (%) | Mgt | 3.146 | 0.005 | 2.751 | 0.072 | 1.095 | 0.341 |
|  | Depth | 2.125 | 0.105 | 1.288 | 0.287 | 0.281 | 0.839 |
|  | Mgt x Depth | 0.410 | 0.989 | 0.286 | 0.941 | 0.481 | 0.820 |
| Clay (%) | Mgt | 1.432 | 0.199 | 4.039 | 0.023 | 0.285 | 0.753 |
|  | Depth | 4.033 | 0.010 | 24.829 | <0.001 | 2.128 | 0.106 |
|  | Mgt x Depth | 0.940 | 0.542 | 0.081 | 0.998 | 0.039 | 1.000 |
| Bulk density (Mg/m^3^) | Mgt | 4.243 | <0.001 | 4.656 | 0.013 | 0.280 | 0.757 |
|  | Depth | 1.052 | 0.373 | 1.127 | 0.345 | 4.741 | 0.005 |
|  | Mgt x Depth | 0.873 | 0.625 | 1.101 | 0.373 | 1.901 | 0.095 |

Table A3: Effects of land management and soil depth on soil texture and bulk density (mean ± standard deviation).

| Site | Factor | | Sand (%) | Silt (%) | Clay (%) | Bulk density (Mg/m^3^) |
| --- | --- | --- | --- | --- | --- | --- |
| Chitedze | Management | 1 | 66 ± 3a | 9 ± 2ab | 25 ± 3 a | 1.47 ± 0.11a |
|  |  | 2 | 68 ± 5a | 9 ± 2ab | 23 ± 5a | 1.34 ± 0.11b |
|  |  | 3 | 67 ± 4a | 9 ± 3ab | 24 ± 4a | 1.31 ± 0.08b |
|  |  | 4 | 66 ± 2a | 10 ± 2b | 24 ± 2a | 1.32 ± 0.09b |
|  |  | 5 | 68 ± 2a | 8 ± 2a | 24 ± 1a | 1.37 ± 0.14ab |
|  |  | 6 | 67 ± 3a | 8 ± 3a | 25 ± 2a | 1.41 ± 0.10ab |
|  |  | 7 | 67 ± 3a | 8 ± 3ab | 25 ± 2a | 1.32 ± 0.11b |
|  |  | 8 | 67 ± 3a | 9 ± 2ab | 24 ± 2a | 1.38 ± 0.08ab |
|  | Depth (cm) | 0-5 | 69 ± 4a | 8 ± 3a | 23 ± 4a | 1.37 ± 0.09a |
|  |  | 5-10 | 68 ± 3ab | 8 ± 2a | 24 ± 2ab | 1.36 ± 0.12a |
|  |  | 10-20 | 67 ± 3b | 8 ± 2a | 25 ± 3b | 1.38 ± 0.13a |
|  |  | 20-30 | 65 ± 3b | 10 ± 2a | 25 ± 2b | 1.34 ± 0.11a |
|  |  |  |  |  |  |  |
| Mwansambo | Management | 1 | 67 ± 8a | 8 ± 2a | 25 ± 7a | 1.46 ± 0.12a |
|  |  | 2 | 72 ± 5b | 6 ± 2a | 22 ±5b | 1.47 ± 0.09a |
|  |  | 3 | 70 ± 6ab | 7 ± 2a | 23 ± 5bc | 1.39 ± 0.08b |
|  | Depth (cm) | 0-5 | 76 ± 4a | 7 ± 2a | 17 ± 4a | 1.46 ± 0.11a |
|  |  | 5-10 | 70 ± 6b | 8 ± 2a | 22 ± 5b | 1.42 ± 0.08a |
|  |  | 10-20 | 66 ± 4bc | 7 ± 3a | 27 ± 4c | 1.43 ± 0.11a |
|  |  | 20-30 | 66 ± 6c | 6 ± 2a | 28 ± 4c | 1.47 ± 0.11a |
|  |  |  |  |  |  |  |
| Lemu | Management | 1 | 77 ± 8a | 5 ± 2a | 18 ± 7a | 1.43 ± 0.13a |
|  |  | 2 | 79 ± 7a | 4 ± 2a | 17 ± 6a | 1.44 ± 0.13a |
|  |  | 3 | 79 ± 8a | 5 ± 3a | 16 ± 6a | 1.41 ± 0.14a |
|  | Depth (cm) | 0-5 | 79 ± 5a | 5 ± 2a | 16 ± 4a | 1.40 ± 0.09ab |
|  |  | 5-10 | 81 ± 6a | 5 ± 1a | 14 ± 5a | 1.51 ± 0.14b |
|  |  | 10-20 | 77 ± 9a | 5 ± 2a | 18 ± 6.9a | 1.44 ± 0.13ab |
|  |  | 20-30 | 76 ± 9a | 5 ± 2a | 19 ± 7a | 1.36 ± 0.14a |

Table A4: Effects of land management and soil depth on soil hydraulic properties (mean ± standard deviation).

| Site | Factor | | Total porosity (%) | Transmission pores (%) | Coarse storage pores (%) | Fine storage pores (%) | Residual pores (%) | K_sat_ (cm/min) | PAWC (%) | Air exchange capacity (%) |
| --- | --- | --- | --- | --- | --- | --- | --- | --- | --- | --- |
| Chitedze | Mgt | 1 | 30.54 ± 6a | 5.52 ± 2.2a | 6.98 ± 2.6a | 5.50 ± 2.1a | 12.54 ± 0.5a | 0.07 ± 0.03a | 7.94 ± 0.8a | 11.21 ± 6.3a |
|  |  | 2 | 44.25 ± 7b | 7.44 ± 2.2bcd | 6.86 ± 1.2a | 11.53 ± 1.9bc | 18.43 ± 2.6bc | 0.23 ± 0.11bc | 13.41 ± 2.4b | 13.03 ± 5.2a |
|  |  | 3 | 45.46 ± 4b | 7.91 ± 2.3cd | 7.16 ± 0.8a | 11.65 ± 1.5bc | 19.74 ± 2.1c | 0.22 ± 0.09bc | 13.45 ± 1.8b | 13.91 ± 3.3a |
|  |  | 4 | 44.91 ± 6b | 9.14 ± 2.3d | 6.32 ± 1.0a | 10.19 ± 1.7b | 19.26 ± 2.4bc | 0.29 ± 0.23c | 12.67 ± 1.4b | 13.74 ± 3.9a |
|  |  | 5 | 41.07 ± 8b | 7.12 ± 2.3abc | 6.44 ± 1.5a | 10.49 ± 2.2bc | 17.02 ± 3.4bc | 0.26 ± 0.17bc | 11.72 ± 2.9b | 12.84 ± 5.5a |
|  |  | 6 | 39.40 ± 9b | 6.09 ± 3.0ab | 6.26 ± 1.4a | 10.67 ± 2.7bc | 16.37 ± 3.2bc | 0.18 ± 0.04bc | 12.22 ± 2.9b | 11.38 ± 4.7a |
|  |  | 7 | 44.28 ± 8b | 8.67 ± 2.5cd | 6.63 ± 1.3a | 10.78 ± 2.1bc | 18.20 ± 3.3bc | 0.24 ± 0.09bc | 12.84 ± 2.3b | 13.90 ± 3.8a |
|  |  | 8 | 43.31 ± 6b | 5.61 ± 1.7a | 6.92 ± 1.1a | 12.57 ± 2.0c | 18.20 ± 2.5bc | 0.24 ± 0.11bc | 14.09 ± 3.0b | 11.54 ± 3.9a |
|  | Depth (cm) | 0-5 | 42.05 ± 8a | 6.41 ± 1.5a | 6.45 ± 1.2a | 10.77 ± 2.8a | 18.41 ± 3.7a | 0.28 ± 0.18a | 12.62 ± 3.0a | 11.64 ± 3.4a |
|  |  | 5-10 | 41.46 ± 9a | 7.71 ± 2.2b | 6.62 ± 1.4a | 10.07 ± 3.4a | 17.07 ± 3.0a | 0.27 ± 0.15a | 12.19 ± 3.2a | 13.06 ± 4.8a |
|  |  | 10-20 | 41.60 ± 8a | 7.80 ± 3.5b | 6.46 ± 1.2a | 10.29 ± 2.5a | 17.05 ± 3.1a | 0.16 ± 0.07b | 11.94 ± 2.5a | 13.22 ± 5.2a |
|  |  | 20-30 | 41.99 ± 8a | 6.84 ± 2.7ab | 7.24 ± 1.8a | 10.56 ± 2.5a | 17.35 ± 3.5a | 0.15 ± 0.06b | 12.42 ± 2.7a | 12.86 ± 5.1a |
|  |  |  |  |  |  |  |  |  |  |  |
| Mwansambo | Mgt | 1 | 37.88 ± 6.0a | 5.87 ± 3.2a | 7.0 ± 3.1a | 7.71 ± 2.2a | 17.30 ± 2.5a | 0.02 ± 0.00a | 9.85 ± 1.0a | 11.78 ± 6.6a |
|  |  | 2 | 40.28 ± 3.8ab | 3.50 ± 1.0b | 6.4 ± 1.2a | 10.22 ± 1.8b | 20.19 ± 1.5b | 0.11 ± 0.10b | 12.50 ± 1.5b | 8.29 ± 4.2a |
|  |  | 3 | 43.08 ± 5.9b | 4.16 ± 1.8b | 6.8 ± 1.9a | 11.00 ± 2.4b | 21.11 ± 1.5b | 0.08 ± 0.09b | 13.85 ± 1.9c | 9.05 ± 5.4a |
|  | Depth (cm) | 0-5 | 39.73 ± 5.7a | 3.58 ± 0.9a | 5.58 ± 0.9a | 11.07 ± 2.1a | 19.48 ± 2.6a | 0.15 ± 0.12a | 12.80 ± 2.7a | 7.90 ± 3.1a |
|  |  | 5-10 | 40.17 ± 5.3a | 5.17 ± 1.6b | 6.87 ± 1.8a | 8.53 ± 2.2b | 19.61 ± 1.3a | 0.04 ± 0.02b | 11.95 ± 2.1a | 10.11 ± 5.6a |
|  |  | 10-20 | 41.61 ± 7.9a | 4.30 ± 2.0ab | 8.46 ± 3.1b | 8.83 ± 2.8b | 20.01 ± 1.7a | 0.03 ± 0.04b | 11.86 ± 2.2a | 10.88 ± 7.3a |
|  |  | 20-30 | 40.14 ± 3.8a | 4.98 ± 3.8b | 5.98 ± 1.2a | 10.14 ± 2.3ab | 19.04 ± 3.7a | 0.05 ± 0.06b | 11.67 ± 2.1a | 9.94 ± 5.7a |
|  |  |  |  |  |  |  |  |  |  |  |
| Lemu | Mgt | 1 | 34.53 ± 7.9a | 16.77 ± 5.7a | 5.29 ± 3.3a | 3.34 ± 1.2a | 9.11 ± 0.8a | 0.09 ± 0.09a | 5.30 ± 1.3a | 21.21 ± 9.4a |
|  |  | 2 | 35.94 ± 6.0a | 7.94 ± 3.8b | 6.62 ± 2.1a | 6.47 ± 2.3b | 14.9 ± 2.9b | 0.13 ± 0.08ab | 8.74 ± 1.6b | 13.27 ± 6.9b |
|  |  | 3 | 37.56 ± 5.6a | 7.96 ± 3.7b | 5.73 ± 0.7a | 9.06 ± 1.3c | 14.8 ± 2.3b | 0.16 ± 0.09b | 9.73 ± 1.6c | 13.32 ± 4.9b |
|  | Depth (cm) | 0-5 | 38.23 ± 4.9a | 11.31 ± 6.4ab | 5.68 ± 1.4ab | 6.85 ± 2.6a | 14.38 ± 4.5a | 0.23 ± 0.08a | 8.22 ± 2.4a | 16.19 ± 6.9a |
|  |  | 5-10 | 35.07 ± 6.5a | 10.79 ± 6.9ab | 6.67 ± 2.3b | 6.19 ± 3.3ab | 11.43 ± 2.7b | 0.08 ± 0.06b | 7.68 ± 2.8a | 16.81 ± 9.6a |
|  |  | 10-20 | 36.83 ± 9.2a | 9.32 ± 5.3a | 6.61 ± 3.1b | 6.61 ± 2.7a | 14.29 ± 3.6a | 0.09 ± 0.07b | 8.46 ± 2.7a | 14.84 ± 9.3a |
|  |  | 20-30 | 33.92 ± 4.4a | 12.14 ± 5.9b | 4.56 ± 1.6a | 5.55 ± 2.8b | 11.66 ± 1.2b | 0.09 ± 0.06b | 7.34 ± 1.7a | 15.90 ± 6.9a |

Table A5: Soil carbon (C) in 0-30 cm depth of the study sites in 2011 and 2019.

| Site | Treatment | Soil C (g/kg) in 2011 | Source | Soil C (g/kg) in 2019 | Source |
| --- | --- | --- | --- | --- | --- |
| Chitedze | CP | 15.47 | Ligowe et al., 2017 | 18.82 | This study |
|  | CACR | 22.43 | Ligowe et al., 2017 | 21.73 | This study |
|  |  |  |  |  |  |
| Mwansambo | CP | 12.60 | Cheesman et al., 2016 | 14.23 | This study |
|  | CACI | 13.38 | Cheesman et al., 2016 | 15.87 | This study |
|  |  |  |  |  |  |
| Lemu | CP | 10.65 | Cheesman et al., 2016 | 9.97 | This study |
|  | CAPI | 10.60 | Cheesman et al., 2016 | 10.38 | This study |

C = carbon, CP = conventional practice, CACR = conservation agriculture with maize-cowpea rotation, CACI = conservation agriculture with maize-cowpea intercrop, CAPI= conservation agriculture with maize-pigeon pea intercrop.

**
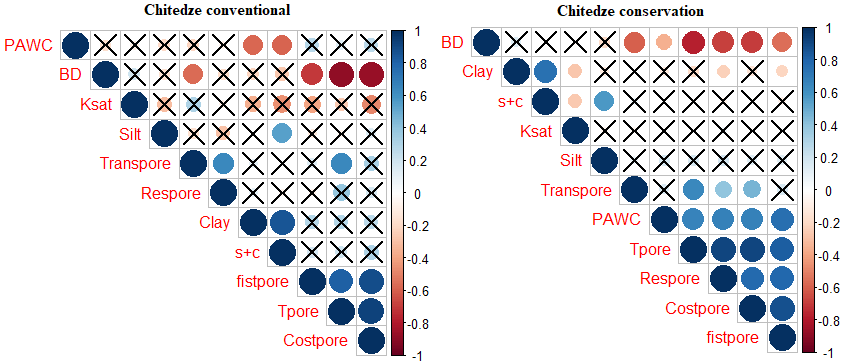
**

Figure A1: Correlation matrix of soil particle size, pore size, hydraulic conductivity and water retention in conventional and conservation plots in Chitedze. Circles = correlation coefficient, Crossed circles = non-significant correlation, Blue circles = positive correlation, Red circles = negative correlation, PAWC = plant available water capacity, BD = bulk density, Ksat = saturated hydraulic conductivity, Transpore = transmission pores, Respore = Residual pores, s+c = silt and clay, fistpore = fine storage pores, Tpore = total porosity, Costpore = Coarse storage pores.

**
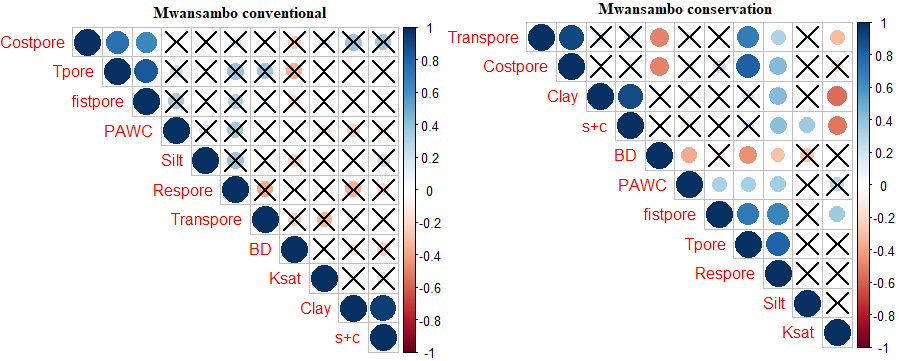
**

Figure A2: Correlation matrix of soil particle size, pore size, hydraulic conductivity and water retention in conventional and conservation plots in Mwansambo. Circles = correlation coefficient, Crossed circles = non-significant correlation, Blue circles = positive correlation, Red circles = negative correlation, PAWC = plant available water capacity, BD = bulk density, Ksat = saturated hydraulic conductivity, Transpore = transmission pores, Respore = Residual pores, s+c = silt and clay, fistpore = fine storage pores, Tpore = total porosity, Costpore = Coarse storage pores.

**
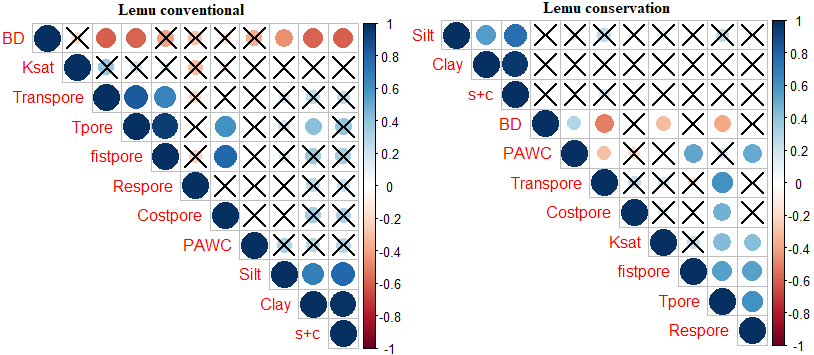
**

Figure A3: Correlation matrix of soil particle size, pore size, hydraulic conductivity and water retention in conventional and conservation plots in Lemu. Circles = correlation coefficient, Crossed circles = non-significant correlation, Blue circles = positive correlation, Red circles = negative correlation, PAWC = plant available water capacity, BD = bulk density, Ksat = saturated hydraulic conductivity, Transpore = transmission pores, Respore = Residual pores, s+c = silt and clay, fistpore = fine storage pores, Tpore = total porosity, Costpore = Coarse storage pores.
